# Supplementary figures and images for: Urban Scaling of Cities in the Netherlands
Source: PLoS One. 2016 Jan 11;11(1):e0146775. doi: 10.1371/journal.pone.0146775 (PMC4708983; doi:10.1371/journal.pone.0146775)

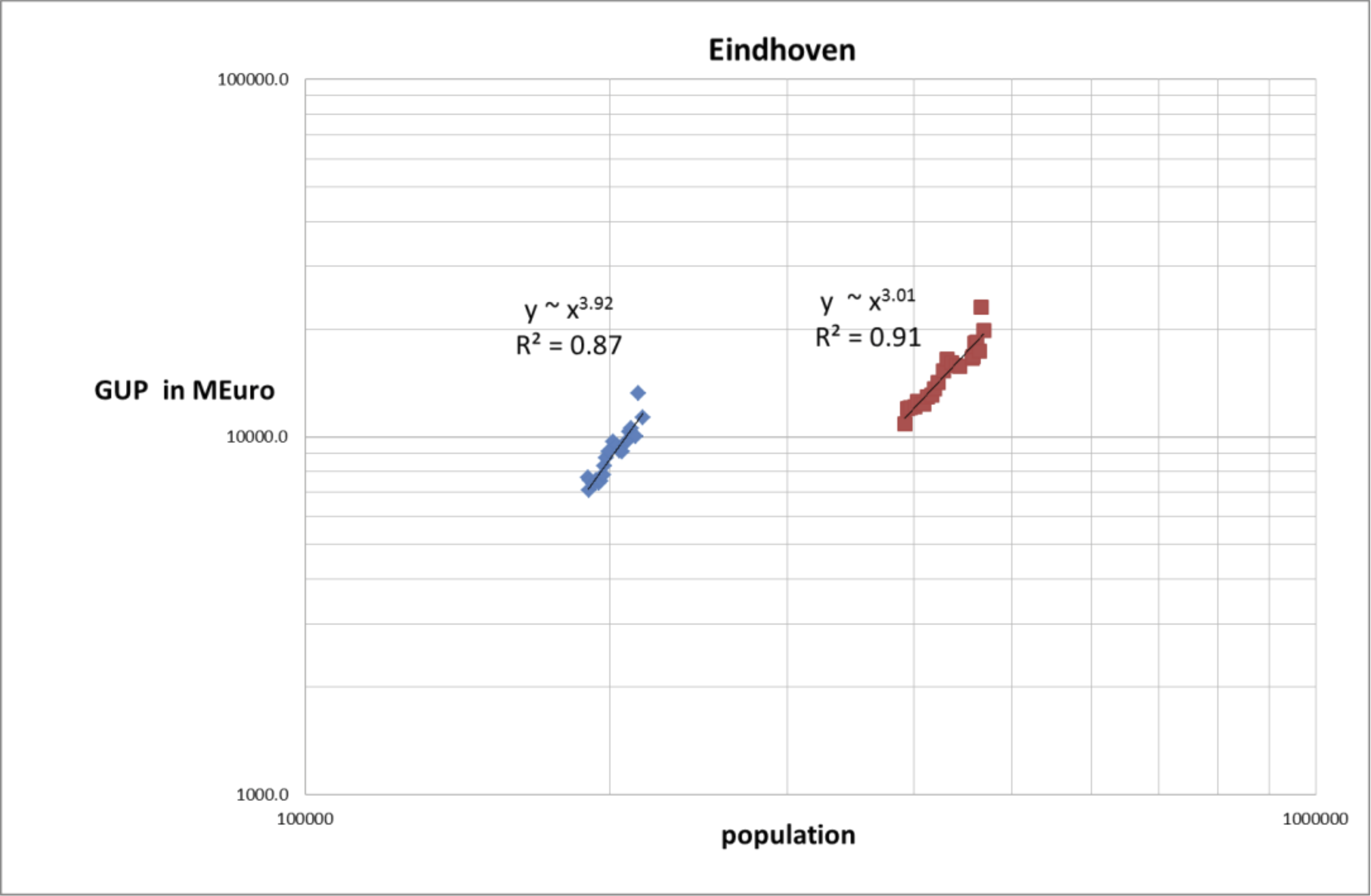

Supplement: S1 Fig — (TIFF) [file pone.0146775.s001.tiff]

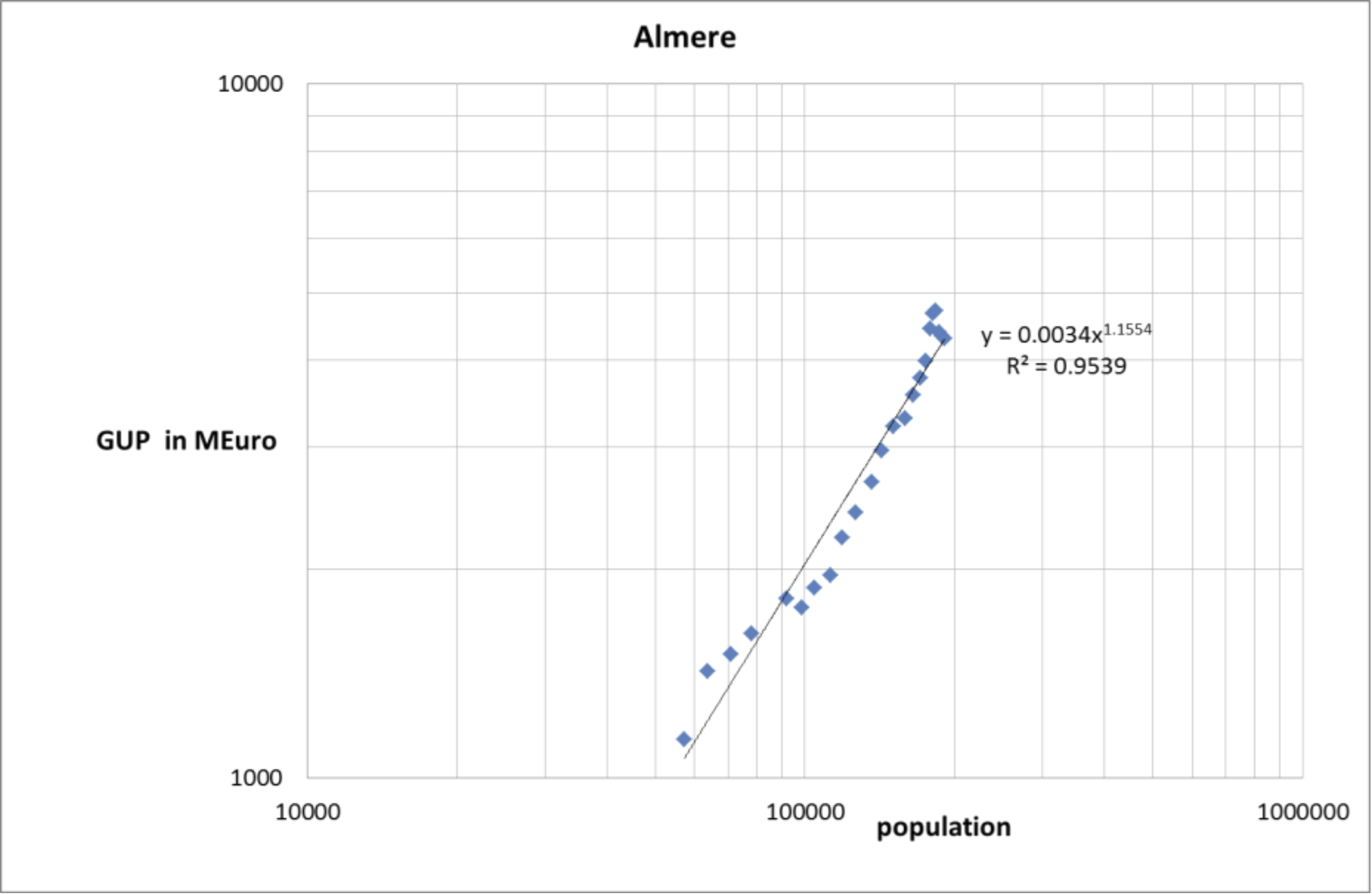

Supplement: S2 Fig — (TIFF) [file pone.0146775.s002.tiff]
