# Supplementary material for: Urban Scaling of Cities in the Netherlands
Source: PLoS One. 2016 Jan 11;11(1):e0146775. doi: 10.1371/journal.pone.0146775 (PMC4708983; doi:10.1371/journal.pone.0146775)
Supplement: S1 Text — (DOCX) [file pone.0146775.s004.docx]

S1 Text

Time-dependent analysis

Generally one can expect that, because of the continuous reinforcing of wealth in a country, the gross urban product of cities will increase slowly as a function of time (average annual rate in the Netherlands of about 2.0 per cent in 1987-2012). In the case of cities with a slow increase of population, this situation will be characterized by an ‘artificially’ very large power-law exponent for the gross urban product as a function of population, as explained mathematically at the end of this section. As an example we show in S1 Fig the scaling of the gross urban product with population of Eindhoven, one of the major industrial centers (Philips, ASML) in the Netherlands. In the period covered by our study (1988-2013) the population of the city (municipality) of Eindhoven (around 200,000 inhabitants) increased with 14 percent and the population of the Eindhoven urban area (around 450,000 inhabitants) with 21 per cent. The GUP (inflation corrected, index 2013) however increased in the same period for the city with 60 percent and for the urban area with 82 percent. As a consequence, we find very high exponents: 3.92 for the city and 3.01 for urban area. Thus it is clear that for cities with a small increase in population the measurement of the scaling behavior of socioeconomic variable with population and resulting exponents is meaningless.

However, for cities that have grown rapidly in the covered time period, the scaling has lower exponents. We present as an example Almere, the new city in the Amsterdam urban area that more than tripled in population from 60,000 in 1988 to about 200,000 now. We find a scaling power-law exponent of 1.16 for the gross urban product, see S2 Fig. This value is in good agreement with those previously found in urban scaling studies and with the expected GUP value for doubling of population as presented in Fig 1 (in the main text). In this case of a rapidly growing city, it is interesting that we find an exponent of 1.43 for the number of jobs. Time analysis of the ratio of GUP and number of jobs shows that the number of jobs increased more rapidly than the GUP. This means a decreasing added value for the more recent jobs. In Section ‘Residual Analysis of the 50,000+ cities’ (in the main text) further evidence is found that this new city underperforms considerably as compared to other cities, in agreement with earlier socio-economic studies of cities in the Netherlands.

Other fast growing cities show similar and even higher power-law exponents. These fast growing cities are characterized by a typical residential role in the beginning (most of these fast growing cities are within the larger urban areas of major cities). But as these cities became larger they started to attract business companies and thus reinforced their socioeconomic position in a relatively great pace, often in a situation where the population was not growing that rapidly anymore. So also in these cases, the high superlinear exponents can be explained, at least a part of it, by the similar mathematical model as discussed here below.

We conclude that a time-dependent analysis of cities indeed reveals the expected scaling behavior, but only in the case of a substantial increase of population, for instance rapidly growing new cities. For slowly growing cities with a relative strong increase of GUP we find very high exponents, as illustrated by the Eindhoven case. It illustrates the difficulty to investigate the scaling behavior with time series data.

To conclude this section, we present a mathematical explanation of large power-law exponents. Suppose we have a slowly growing gross urban product

${G\left( t \right)\sim e}^{\alpha t}$ (1)

and an even slower growing population

$${P\left( t \right)\sim e}^{\beta t}, \beta<\alpha$$

 (2)

Say we are interested in the gross urban product as a function of P, then from the necessary condition

$\int G\left( t \right)dt =\int G(P)dP$ (3)

and using Eq. 2 from which follows $t \sim\frac{1}{\beta} lnP$ we find

$G\left( P \right)=G\left( t \right)\frac{dt}{dP} \sim\frac{e^{\alpha t}}{\beta e^{\beta t}}= \frac{1}{\beta} e^{\left( \alpha-\beta\right)\left( \frac{1}{\beta} \right)lnP}= \frac{1}{\beta}P^{(\frac{\alpha}{\beta}-1)}$ (4)

which is a power-law with exponent γ = (α/β – 1).

It is now obvious from Eq. 4 that in the case of a very slow increase in population (small β, 0.006 in the Eindhoven case) and a general increase of the gross urban product with a larger exponent (α, typically 0.020), the power-law exponent of the gross urban product as a function of population will be considerably higher than 1, we find an exponent of around 3, as is indeed the case for Eindhoven.
